# Redesigning an antibody H3 loop by virtual screening of a small library of human germline-derived sequences

**DOI:** 10.1038/s41598-021-00669-w

**Published:** 2021-11-01

**Authors:** Christopher R. Corbeil, Mahder Seifu Manenda, Traian Sulea, Jason Baardsnes, Marie-Ève Picard, Hervé Hogues, Francis Gaudreault, Christophe Deprez, Rong Shi, Enrico O. Purisima

**Affiliations:** 1 Human Health Therapeutics Research Centre, National Research Council Canada, 6100 Royalmount Avenue, Montreal, QC H4P 2R2 Canada; 2 Département de Biochimie, de Microbiologie et de Bio-Informatique, PROTEO, and Institut de Biologie Intégrative et des Systèmes (IBIS), Université Laval, Pavillon Charles-Eugène-Marchand, Quebec City, QC G1V 0A6 Canada; 3 Biochemistry Department, McGill University, 3655 Promenade Sir William Osler, Montreal, QC H3G 1Y6 Canada

**Keywords:** Protein design, Molecular modelling

## Abstract

The design of superior biologic therapeutics, including antibodies and engineered proteins, involves optimizing their specific ability to bind to disease-related molecular targets. Previously, we developed and applied the Assisted Design of Antibody and Protein Therapeutics (ADAPT) platform for virtual affinity maturation of antibodies (Vivcharuk et al. in PLoS One 12(7):e0181490, 10.1371/journal.pone.0181490, 2017). However, ADAPT is limited to point mutations of hot-spot residues in existing CDR loops. In this study, we explore the possibility of wholesale replacement of the entire H3 loop with no restriction to maintain the parental loop length. This complements other currently published studies that sample replacements for the CDR loops L1, L2, L3, H1 and H2. Given the immense sequence space theoretically available to H3, we focused on the virtual grafting of over 5000 human germline-derived H3 sequences from the IGMT/LIGM database increasing the diversity of the sequence space when compared to using crystalized H3 loop sequences. H3 loop conformations are generated and scored to identify optimized H3 sequences. Experimental testing of high-ranking H3 sequences grafted into the framework of the bH1 antibody against human VEGF-A led to the discovery of multiple hits, some of which had similar or better affinities relative to the parental antibody. In over 75% of the tested designs, the re-designed H3 loop contributed favorably to overall binding affinity. The hits also demonstrated good developability attributes such as high thermal stability and no aggregation. Crystal structures of select re-designed H3 variants were solved and indicated that although some deviations from predicted structures were seen in the more solvent accessible regions of the H3 loop, they did not significantly affect predicted affinity scores.

## Introduction

Antibodies can target a wide variety of foreign pathogens due to the diversity found within their binding region, known as the complementarity-determining region (CDR). This exquisite versatility has led to the widespread development of antibodies by all sectors of biomedical research community. Antibodies are excellent templates to design and engineer novel therapeutics due to their modular structure. New antibodies can be found and optimized through numerous experimental techniques^1^, such a phage^2,3^ or yeast display^4^ and animal immunization^5,6^. Complementary to these experimental methods, structure-based computational techniques have increasingly been used to guide the design of novel antibodies with desired properties and/or functionality^7–16^. Of particular interest is the application of these techniques for affinity maturation^17–22^. These initial forays into antibody affinity maturation allow only amino-acid mutations with no changes in sequence length. Typically, a crystal structure of the antibody–antigen complex forms the basis for sequence optimization. Most methods involve optimizing the side chain of the mutant(s) followed by predicting the relative change in binding affinity using a scoring function. Clark et al*.*^21^ were the first that demonstrated that computational techniques can improve antibody affinity upwards of tenfold using a 4-point mutant. This work was followed by Lippow et al.^22^, where they affinity-matured the anti-lysozyme antibody D44.1 to identify a mutant with 140-fold increased affinity. Work by Sulea et al.^19^ showed that using a consensus over multiple methods can increase the accuracy of relative binding affinity predictions. The group then applied the consensus approach within the Assisted Design of Antibody and Protein Therapeutics (ADAPT) platform to computationally design multiple antibodies with 1- to 4-point mutations resulting in improved affinities upwards of 140-fold^18^.

These simple techniques have now spawned more complex uses such as modulating the specificity of antibodies. Farady et al.^23^ were able to computationally design a species cross-reactive antibody which binds both the human and mouse homologues of an antigen. Sulea et al*.*^24^ computationally designed a pH-selective antibody which can preferentially bind its target at acidic pH of tumors (6.4) relative to physiological pH. These approaches involve the introduction of point mutations with no insertions or deletions and generally assume minimal perturbation of the parent backbone structure. A less conservative approach is to replace or graft whole CDR loops onto the antibody framework. Typically, this involves using known antibody sequences as a starting pool, followed by mixing and matching antibody parts to yield an antibody with novel functionality^11^.

The OptCDR method by Pantazes and Maranas is built on this principle to design CDRs libraries for a defined antigen by sampling and combining CDR canonical clusters^25^. It then refines the sequences and conformations of the CDR loops to create a sequence library. Application of OptCDR led to a library of 50 single-chain Fv (scFv) variants predicted to bind a FLAG tetrapeptide (DYKD), 4 of which had nanomolar affinity^26^, yet were less stable than the parent framework antibody that was used for the computational screen. Maranas followed up this work with OptMAVEn which extended the OptCDR approach to optimizing entire Fv domains^27,28^. Inspired by the natural V(D)J recombination in the immune system, OptMAVEn divides the antibody structure into a pre-CDR3, CDR3 and post-CDR3 segments for both the heavy and light chains selected from their MAPs database^29^, followed by in silico affinity maturation and refinement. OptMAVEn was used to design 5 antibodies targeting a 12-mer peptide starting from a crystal structure of a parental scFv antibody^30^. These 5 designs were significantly different from the parental antibody, 3 of which had nanomolar affinity, yet none had stronger affinities than the parental scFv.

Similar to OptCDR/OptMAVEn is AbDesign developed by Fleishman and co-workers^31^. Whereas OptMAVEn focused on splicing antibodies similar to V(D)J recombination, AbDesign focused of splicing antibodies along structurally conserved regions in the framework between CDR2 and CDR3 in both the heavy and light chains^32^. AbDesign was validated by de novo designing weak-affinity antibodies for human insulin and acyl-carrier protein 2 (ACP2)^32^. Thus, 114 designs for human insulin were selected resulting in one binder which underwent maturation to a binding affinity of 50 nM. Similarly, 79 designs were selected for ACP2 resulting in 2 binders followed by maturation yielding antibodies with affinities in the range of 50–100 nM.

Similar in spirit to AbDesign is the Rosetta Antibody Design (RAbD) development by Dunbrack and coworkers^33^. Like AbDesign, RAbD uses the Rosetta modeling toolkit to design and optimize antibodies by splicing CDRs into an antibody framework followed by sequence design in additional to novel features such as optimizing the non-CDR DE loop. They optimized 2 antibodies targeting hyaluronidase and HIV gp120 by swapping out the native CDR loops for new ones taken from a pre-existing antibody–antigen complex structure. From the 30 designs selected against hyaluronidase, 17 had some binding in the μM range while 3 others achieved better affinities than the parent with upwards of a 12-fold improvement. Out of the 27 designs selected against gp120, 6 were able to bind one or more of the seven gp120 variants with only one of those achieving better binding than the parent antibody.

**Figure 1:**
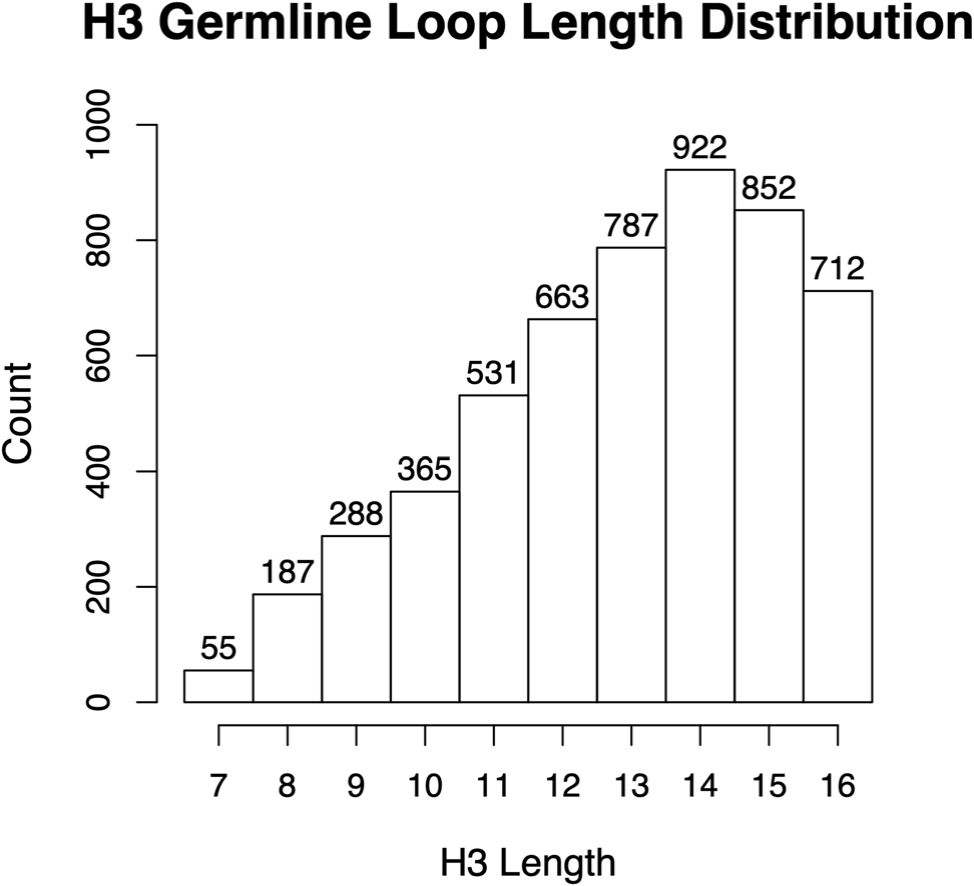
Length distribution of the H3 loop library used in this study.

In this study, we focused on re-engineering the hypervariable H3 of existing antibodies, owing to its role as a major determinant of antigen binding affinity and specificity. Thus, starting from a crystal structure of the parental antibody–antigen complex, we selected new CDRH3 sequences and kept sequences for the other CDR loops and the framework region unchanged. We used germline-derived V(D)J rearranged H3 sequences from known human antibodies available from IMGT/LIGM-DB^34^. We selected a range of H3 sequence lengths from 7 to 16 residues long (Fig. 1). Although, this set amounts to a relatively modest number of sequences (10^3^–10^4^), it is an order of magnitude larger than the affinity-matured H3 sequences available in the PDB (10^2^–10^3^)^35–38^. Using actual observed H3 sequences rather than artificial ones may improve antibody developability profile in terms of stability, aggregation and immunogenicity. The caveat is that the IMGT-based sequences require generating structural models for the H3 loops in complex with the antigen, which brings its own set of challenges^12,39–41^.

## Results

### Application to bH1-VEGF as proof-of-concept

**Figure 2:**
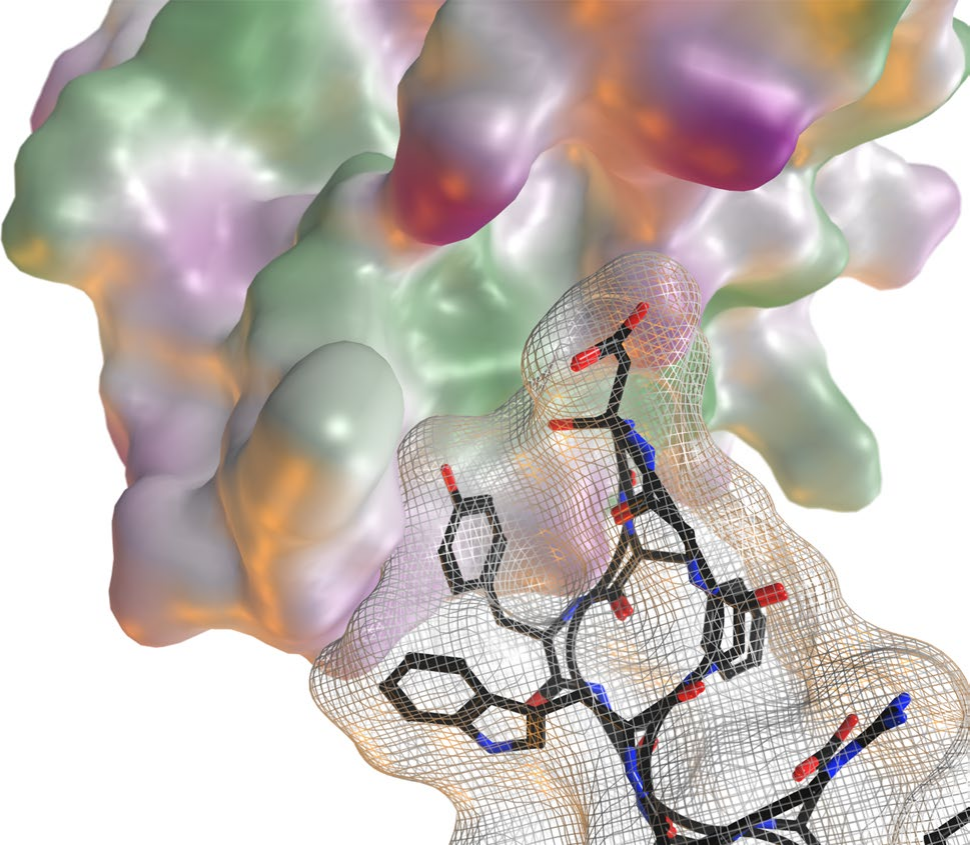
Examination of the bH1 epitope on VEGF (PDB ID: 3BDY) reveals empty space close to H3. The VEGF antigen is shown as a surface, the bH1 antibody is shown with a light-grey mesh/ribbon, except the H3 which is shown with black tube and ball-and-stick residues.

We have previously used the bH1-VEGF antibody–antigen pair (PDB ID: 3BDY) as a test case for affinity maturation using the ADAPT protocol, which involved introducing point mutations^18^. In the current study, we used the same system to evaluate the feasibility of swapping in H3 loop replacements. This system has some properties that make it suitable as proof-of-concept test case for this study. The H3 loop of the parental antibody contributes only around 20% to the overall calculated binding affinity according to the SIE scoring function, which suggests that the canonical CDR loops may be sufficient to retain some level of binding at the same epitope even with only modest contribution from the grafted H3 loop. Also, when examining the structural epitope of the parental H3 loop, a significant amount of empty space is present, suggesting that it may be possible to fit a variety of H3 loop lengths within this area (Fig. 2). We do not expect our small library of H3 loop sequences to necessarily contain affinity-matured loops for our target. The hope is to identify viable new H3 loops that could provide is a new starting point for subsequent affinity maturation, augmenting the sequence space explored by point mutations on the parental structure alone.

#### H3 library

5362 sequences of H3 loops with lengths from 7–16 residues were extracted from the IMGT/LIGM-DB^42^ in 2016 with close to 70% of the loops having a length of 12 amino-acid residues or longer (IMGT definition of CDR boundaries) (see Fig. 1). This was achieved by searching the IMGT/LIGM-DB website for all human IGH productive rearranged sequences in which the CDR3-IMGT is annotated. This set includes germline rearranged sequences as well as affinity-matured sequences. Sequences containing cysteines were removed.

#### H3 loop grafting

Over 5000 H3 sequences were virtually grafted into the 3BDY template system (bH1-Fv binding VEGF) using the protocol described in the “Materials and methods” section. Grafting of H3 loops from the sequence library required prediction of their loop conformation. Moreover, most of the H3 library is comprised of longer loops (> 8 amino-acids) and therefore can be challenging for accurate structure prediction^39,43^. Unlike the other five CDR loops, there are no well-defined canonical structures for the H3 loop. However, sequence motifs do exist that can be used to limit the conformational space of the H3 loop. For example, the H3 loop can exhibit an “kinked” or “extended” conformation depending on its sequence and structural environment^44^. In fact, RosettaAntibody uses this information as a filter to eliminate conformations which do not meet this requirement based on the input sequence. In addition, North et al*.*^45^ have suggested a structural clustering scheme based on H3 sequences focusing on the stem region of the loop (the first 3 residues and the last 4 residues according to IGMT definition^46^). Using this information as a starting point for H3 loop prediction can artificially shorten the loop length needed to be conformationally sampled. In effect using these “shorter” loops enabled the structural prediction our library (Fig. 1) more tractable.

**Figure 3:**
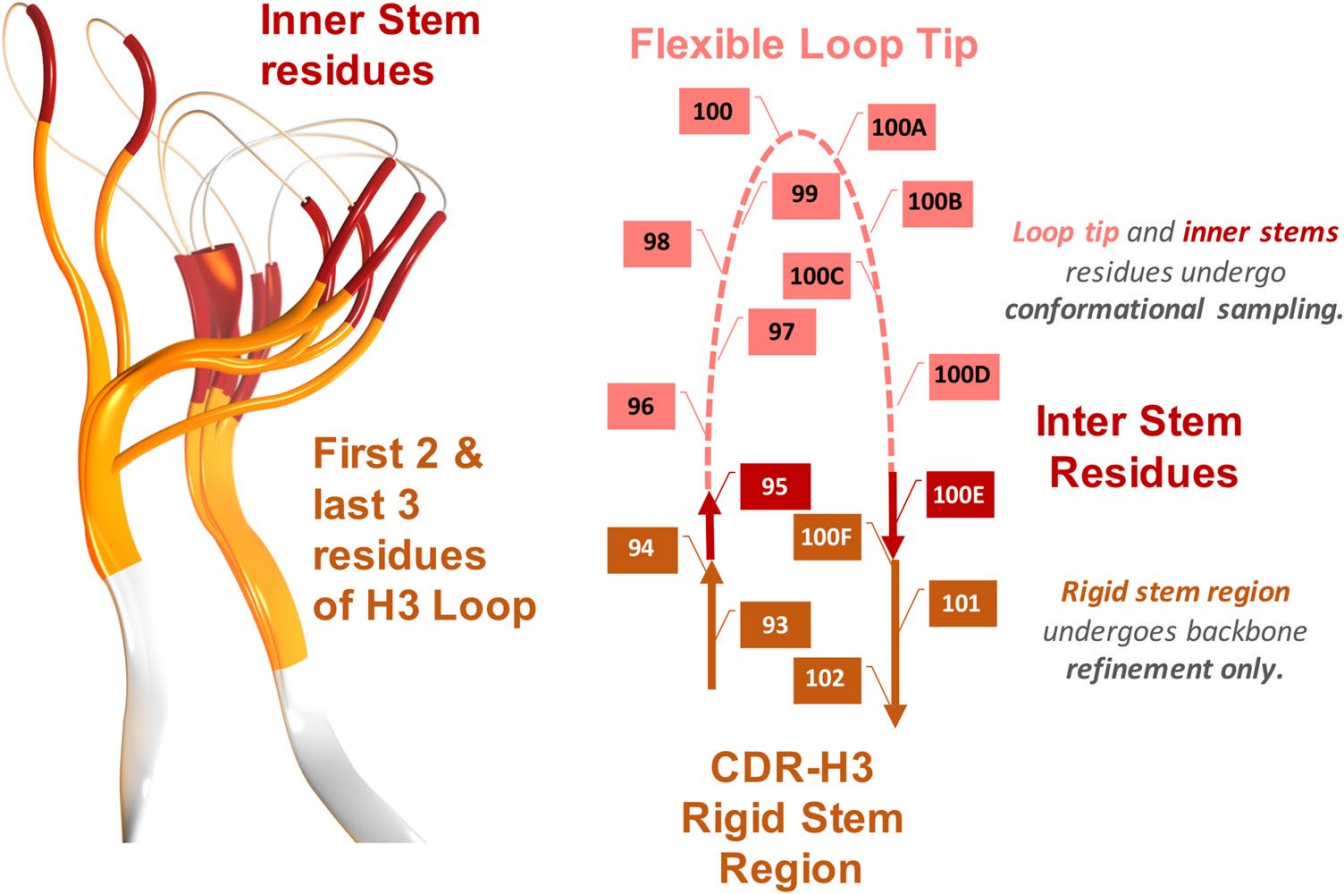
Definition of the H3 stem region for template matching. N- and C-terminal regions of the stem as defined by North et al. are rendered as cartoon, with the remaining inner H3 loop region that needs to be sampled shown as thin line. Larger structural variations for inner ends (red) of the stem led to a shortened stem (yellow) to be used in our approach.

In situations where multiple stem templates matched a given H3 stem sequence, all matching stem templates were used. Matching 3D-stem-templates were then grafted into the parental antibody–antigen structure. The loop was then mutated to the correct residue identities for the entire loop using SCWRL to create a stem target. 3D-stem-templates of H3 loops with longer than 7 residues exhibited larger structural variations of the inner ends, 1 position on the N-stem and 1 position on the C-stem (see Fig. 3).

This therefore required that stem templates be shortened to only include the first 2 and last 3 residues of the H3 sequence (Table S1) to allow for sampling of these inner ends. After the initial grafting, a loop ensemble was generated with a multi-stage refinement protocol as described in the “Materials and methods” section.

#### H3 affinity ranking

**Figure 4:**
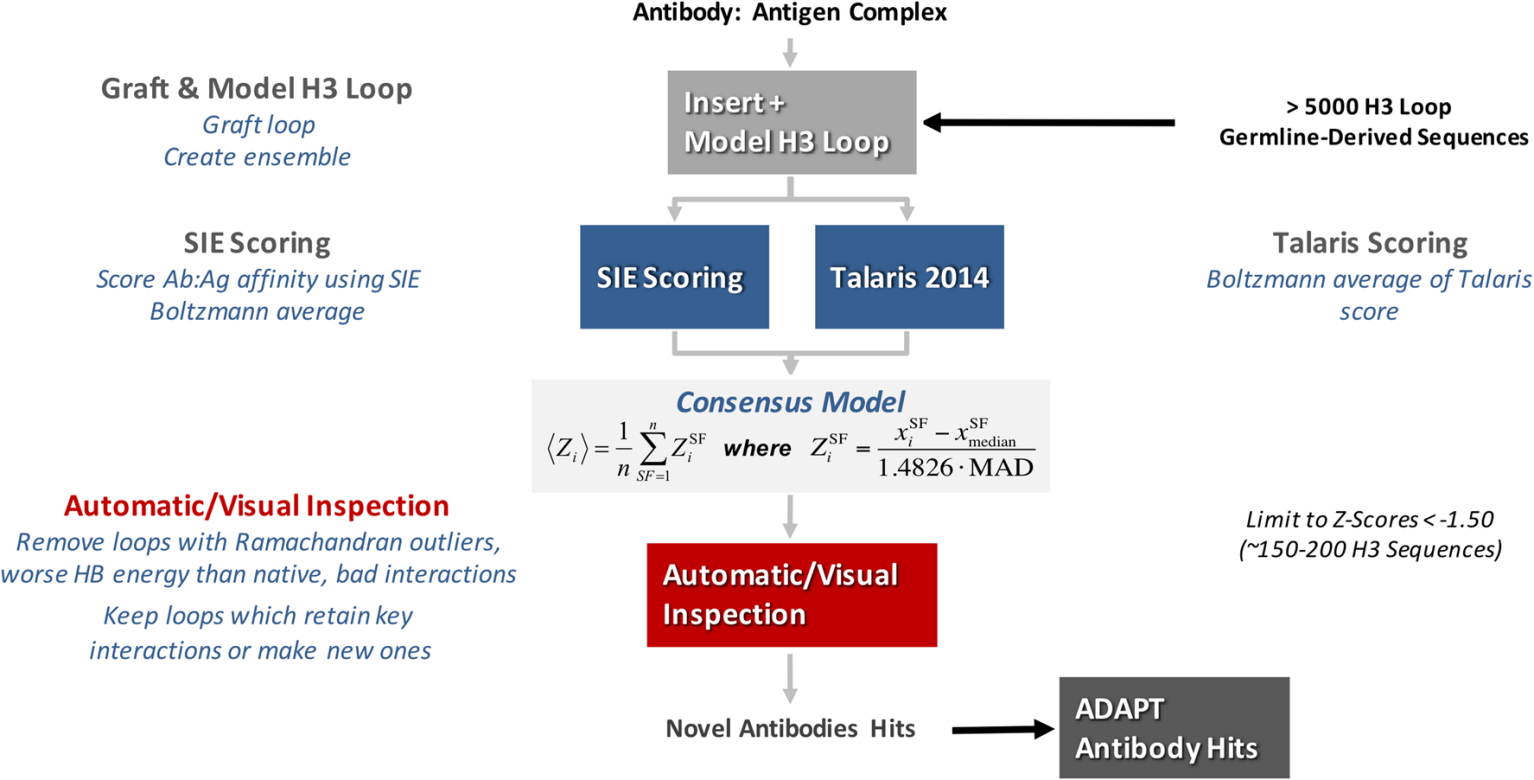
Antibody–antigen binding affinity scoring protocol. The antibodies with their modeled H3 loop ensembles are scored for antigen binding using SIE and Talaris energies combined into a consensus score. This model is used to rank the best designs. Visual inspection is then used to remove candidates that have obvious flaws in internal geometry and/or intermolecular interactions.

A consensus *Z*-score of the SIE and Talaris energy scores was used to predict relative binding affinities (Fig. 4). The Rosetta Talaris total energy score, which includes intermolecular and internal energy terms, was calculated for various conformers in loop ensemble generated by Rosetta. The SIE scores were calculated on AMBER-minimized structures from the same ensemble. A Boltzmann average was used for both SIE and Talaris energy scores, with the Talaris total energy being used to weight the average. The advantage of using these two scoring functions is that SIE captures mainly affinity while Talaris in affect captures the stability of the complex by including some intramolecular terms. We initially observed that in general longer H3 sequences tended to have better energy scores than shorter ones and would out-compete them (see Table S2). Thus, good *Z*-scores computed across a combined pool of sequence lengths would be dominated by longer sequences. This is most likely due to the well-known bias of the scoring functions preferring molecules with a greater number of atoms^47–49^. To overcome this size bias, *Z*-scores were calculated separately for each H3 sequence length. Unless stated otherwise, *Z*-scores are understood in this paper to refer to these length-specific scores. A *Z*-score of −1.5 was used as the cutoff to enable a manageable number of candidates (88 sequences) for manual curation. If we had not calculated separate *Z*-scores for each loop length, no H3 loops with a length less than 13 residues would have passed the cutoff of −1.5 (see Table S2).

To reduce the cost of producing and testing the designed H3 sequences, the selection was filtered further as follows. (1) For each sequence, the best-scoring loop conformation of the ensemble must have no backbone angles in the disallowed region of the Ramachandran map according to ProCheck^50^; (2) the hydrogen bonding energy according to ProPOSE^51^ must not be significantly worse (> 1 kcal/mol) than in the parental loop structure; (3) visual inspection for non-ideal interactions or loop structure. Structural visualization and analysis were done in MOE (Chemical Computing Group). This yielded 16 H3 loops (Table 1), 13 of which were ranked within the top-50 sequences according to *Z*-score.

**Table 1:** Selected H3 sequences for experimental validations.

Design ID	Sequence	H3 length^a^	*Z*-score^b^	Rank^c^		
				TLR	SIE	*Z*-score
Parent	SRWGGDGFYAMDY	13	− 2.55	80	18	1
16_0325	ARGGAVAGTGVYYFDY	16	− 1.71	153	13	41
14_0112	AKGGSSSGPYHFEY	14	− 1.79	296	5	35
14_0472	ARGIAVAGAYYFDY	14	− 2.09	41	36	14
13_0346	ARGGSFYYYYMDV	13	− 1.81	212	181	34
16_0102	AKGWEGTTVTLTPVDY	16	− 1.56	90	105	79
15_0485	ARHGVRGYYYYYMDV	15	− 2.07	33	30	18
14_0905	VRGGYLRDYYGMDV	14	− 1.94	12	249	27
14_0130	AKLGIGYYYYGMDV	14	− 2.46	8	20	3
14_0822	GRSGPRLGMYYFDF	14	− 1.99	68	39	25
12_0327	ARGRKYSSSFDY	12	− 1.60	746	144	68
14_0480	ARGLERSGNYYLDY	14	− 2.08	28	75	15
14_0129	AKLGGQGSYYHFDY	14	− 2.08	72	10	16
16_0460	ARPSGGSRSWLYYFDY	16	− 1.68	5	533	47
14_0490	ARGNEAGYYYGMDV	14	− 1.70	128	92	42
14_0622	ARNGGDSYSGYFQH	14	− 1.70	310	16	43
14_0688	ARSGRDAYNYYFDS	14	− 1.66	56	221	54

^a^ Loop sequence length, in amino-acid residues, according to the IGMT definition^46^.

^b^ *Z*-scores are calculated for the given loop length.

^c^ Ranks for the TLR (Rosetta Talaris) and SIE scores are across the entire library. The *Z*-score value is calculated using a median for a given length. *Z*-scores for all designs are then merged and a rank determined.

One possible concern is that the affinities measured for the new constructs might be due solely to the other 5 CDR loops with no contribution from the designed H3. To address this, a minimal H3 (minH3) that reduces the H3 from 13 residues (bH1 H3 = SRWGGDGFYAMDY) to the 7 stem residues (minH3 = SRWGMDY) was included as a control.

### Experimental determination of relative binding affinities

**Figure 5:**
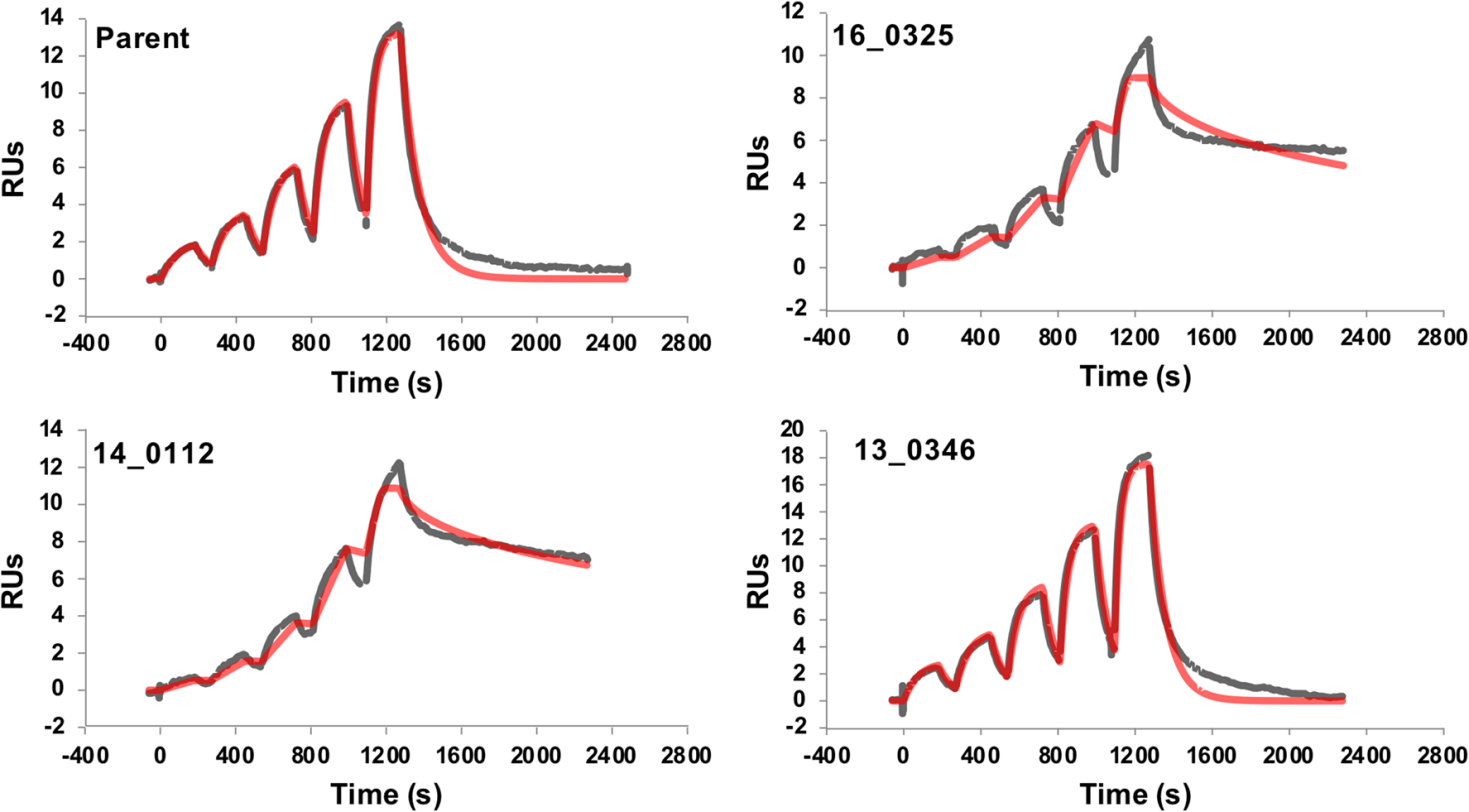
SPR sensorgrams. The red curves represent the global fits of the data to a 1:1 bimolecular interaction model.

These 16 selected sequences along with minH3 were then sent for production, purification, biophysical characterization and SPR measurements of their binding affinities (Fig. 5). All variants behaved well in terms of developability attributes such as thermal stability and lack of aggregation, mirroring the parental bH1 Fab (see Figures S1 and S2). A construct using a 7-residue poly-glycine loop as H3 was also made but was not stable (data not shown). This indicates the necessity of retaining the H3 stem as support for the canonical CDRs. The control, minH3, had a *K*_D_ of 16 μM and was used as the baseline or “residual” binding affinity of a variant due to the other 5 CDR loops. In 13 out of 16 variants (77%) the new H3 loop provided additional binding affinity over the baseline (Table 2). This underscores that this protocol can provide energetically feasible H3 loop replacements. One of the designs exhibits a 200-fold increase in affinity over the minH3 construct. Two other sequences appear to have even stronger affinity, although the fit to the SPR sensorgram using a standard 1:1 Langmuir binding model is somewhat poor for these two (Fig. 5). Ten of the designs had modest affinity improvements ranging from 3 to 15-fold when compared to the minH3 construct. Taking the parental sequence as reference, 2 designs appear to have significant affinity improvements, while 10 designs had modest affinity decreases ranging from 5 to 50-fold, and 3 designs having 500-fold or greater loss in affinity. The 2 best improvers have low sequence identity/similarity and longer length between the H3 designs and the H3 parental sequence.

**Table 2:** Selected H3 sequences for experimental validations.

Design ID	H3 sequence	H3 length^a^	H3 sequence^b^		*K*_D_ (nM)	${K}_{\text{D}}^{\text{Parent}}/{K}_{\text{D}}^{\text{Design}}$	${K}_{\text{D}}^{\text{minH3}}/{K}_{\text{D}}^{\text{Design}}$	T_m_ (C)^d^
			Identity	Similarity				
minH3	SRWGMDY	7	100	100	16,000	0.0063	1.0	
Parent	SRWGGDGFYAMDY	13	100	100	100	1.0	160	77.5
**16_0325**	ARGGAVAGTGVYYFDY	**16**	**31**	**31**	**8**^c^	**13**	**2000**	81.3
14_0112	AKGGSSSGPYHFEY	14	21	29	8^c^	13	2000	81.3
14_0472	ARGIAVAGAYYFDY	14	29	29	59,000	0.0017	0.27	83.4
**13_0346**	ARGGSFYYYYMDV	**13**	**39**	**54**	**80**	**1.3**	**200**	83.5
16_0102	AKGWEGTTVTLTPVDY	16	13	19	50,000	0.002	0.32	76.5
15_0485	ARHGVRGYYYYYMDV	15	27	40	310	0.32	52	78.8
14_0905	VRGGYLRDYYGMDV	14	21	29	5000	0.02	3.2	81.1
**14_0130**	AKLGIGYYYYGMDV	**14**	**29**	**36**	**2800**	**0.04**	**5.7**	76.6
14_0822	GRSGPRLGMYYFDF	14	21	29	> 50,000	< 0.002	< 0.3	81.1
**12_0327**	ARGRKYSSSFDY	**12**	**25**	**33**	**1600**	**0.063**	**10**	79.4
14_0480	ARGLERSGNYYLDY	14	29	36	2720	0.037	5.9	79.7
14_0129	AKLGGQGSYYHFDY	14	29	36	3480	0.029	4.6	75.2
16_0460	ARPSGGSRSWLYYFDY	16	25	25	4500	0.022	3.6	76.3
14_0490	ARGNEAGYYYGMDV	14	21	29	3590	0.028	4.5	79.3
14_0622	ARNGGDSYSGYFQH	14	7.1	14	4970	0.02	3.2	83.3
14_0688	ARSGRDAYNYYFDS	14	14	14	3600	0.028	4.4	86.5

^a^Loop sequence length, in amino-acid residues, according to the IGMT definition^46^.

^b^H3 sequence identity and similarity relative to the parental H3 sequence as determined using MOE (Chemical Computing Group) alignment tools.

Highlighted in bold are the designs structurally characterized by X-ray crystallography.

^c^Poor fit to 1:1 Langmuir binding model.

^d^See DSC thermograms (Figure S2).

### Experimental structure determination for selected designs

Four of the engineered bH1-Fab H3 variants were selected for co-crystallization with the VEGF antigen and X-ray diffraction to confirm that the binding mode did not change upon the redesign of the H3. For a description of the X-ray crystallography methods and an in-depth analysis of the crystal structures see the work of Shi et al*.* (*in preparation*). Here we present a comparison between the crystal structures and predicted models. These were done by superposing the antibody Fv framework backbone atoms of the predicted conformations onto the first refined copy of the crystal structure for each design. RMSD and BRMSD values were calculated between the Talaris energy best-scoring conformation of the H3 loop ensemble and the crystal structure for each variant (Table 3). Using other independently refined crystallographic copies of the antibody–antigen complex structure impacts results minimally (± 0.1 Å in RMSD values). As only the H3 was allowed to move during the conformational search, comparing other features such as canonical CDR loops between predicted and crystal conformations is the same as comparing the crystal structures of the designs to the parental crystal structure.

**Table 3:** Comparison between best scoring H3 loop conformation and crystal structure for 4 designs. PDB Code corresponds to the crystal structure used for the comparison to the predicted loop conformation of the design.

Design ID	PDB code	RMSD (Å)							
		H1	H2	H3	L1	L2	L3	FR (Fv)	Antigen
Parent	3BDY	0.15	0.12	1.47	0.15	0.13	0.38	0.16	0.54
16_0325	7KEZ	1.39	1.93	3.21	1.40	1.03	1.20	0.80	1.75
13_0346	7KF0	1.05	2.11	3.12	0.94	0.89	1.09	0.64	2.62
14_0130	7KF1	1.67	1.84	5.95	1.26	0.42	1.43	0.87	1.83
12_0327	7KF2	1.18	0.65	2.58	1.88	0.73	1.76	0.70	2.44

Design ID	PDB code	BRMSD (Å)							
		H1	H2	H3	L1	L2	CL3	FR (Fv)	Antigen
Parent	3BDY	0.06	0.06	0.83	0.06	0.06	0.07	0.07	0.53
16_0325	7KEZ	0.62	0.74	3.04	0.79	0.62	0.99	0.38	1.56
13_0346	7KF0	0.54	0.87	2.61	0.77	0.50	0.91	0.39	2.52
14_0130	7KF1	0.51	1.13	3.43	0.66	0.35	1.13	0.56	1.66
12_0327	7KF2	0.45	0.51	2.13	0.68	0.41	1.18	0.34	2.24

Best-scoring H3 loop conformation are based on the Talaris total energy. The first copy in the crystal structure was used. IGMT definition was applied for delineation of CDR loops^46^. FR (Fv) is the framework the Fv region. Larger (B)RMSD values calculated for the for the antigen are due to superposition being directed towards one of two binding sites of the VEGF dimer.

**Figure 6:**
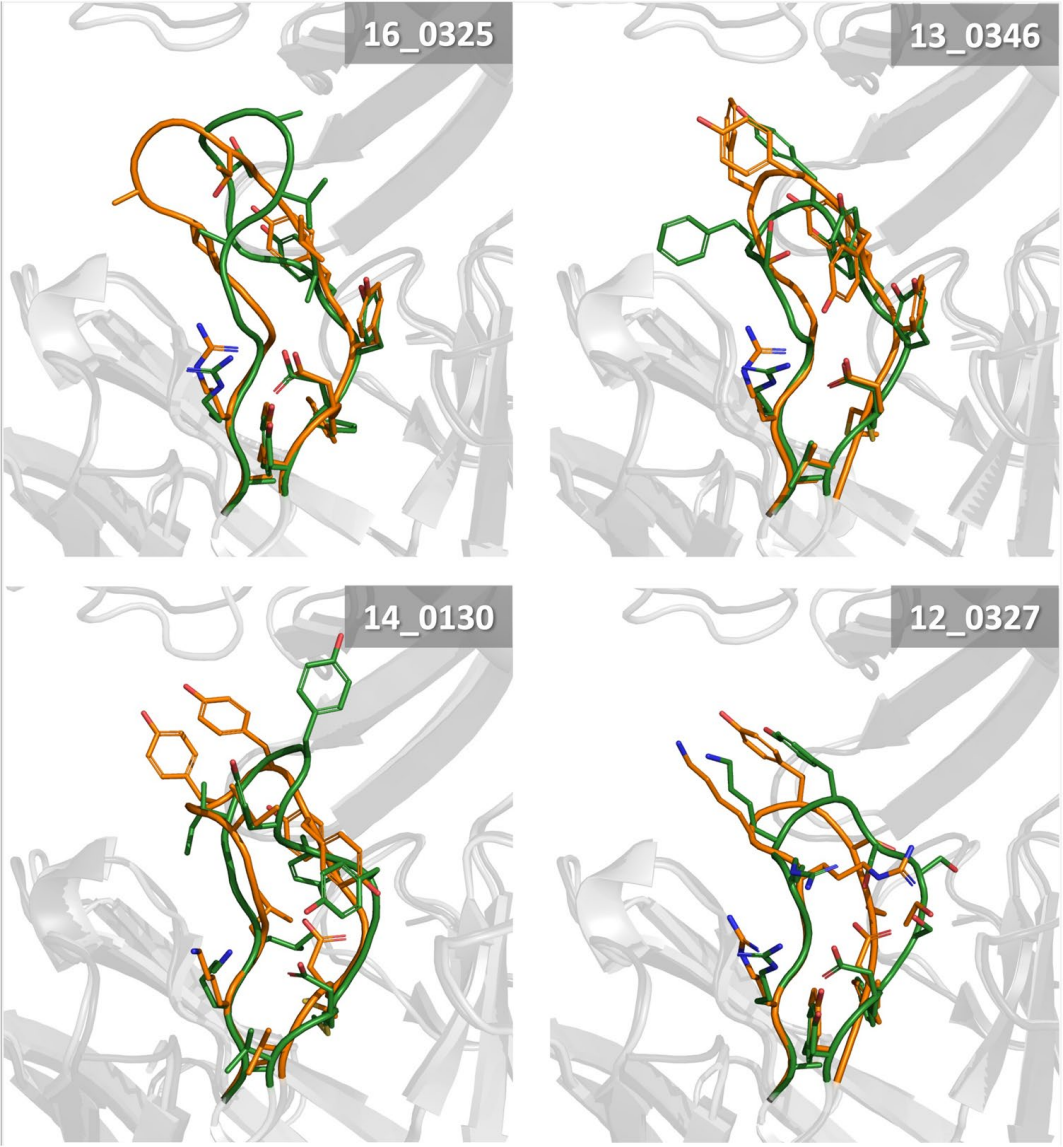
Structural validation of four H3 redesigns. Sequences for design IDs 16_0325, 13_0346, 14_0130 and 12_0327 are shown in Table 3. In light and dark gray are the crystal and predicted structures, respectively, for each prospective design. The experimentally observed conformation of H3 loop is shown in green. The best-scoring Talaris energy-based H3 loop conformation is shown in orange.

**Figure 7:**
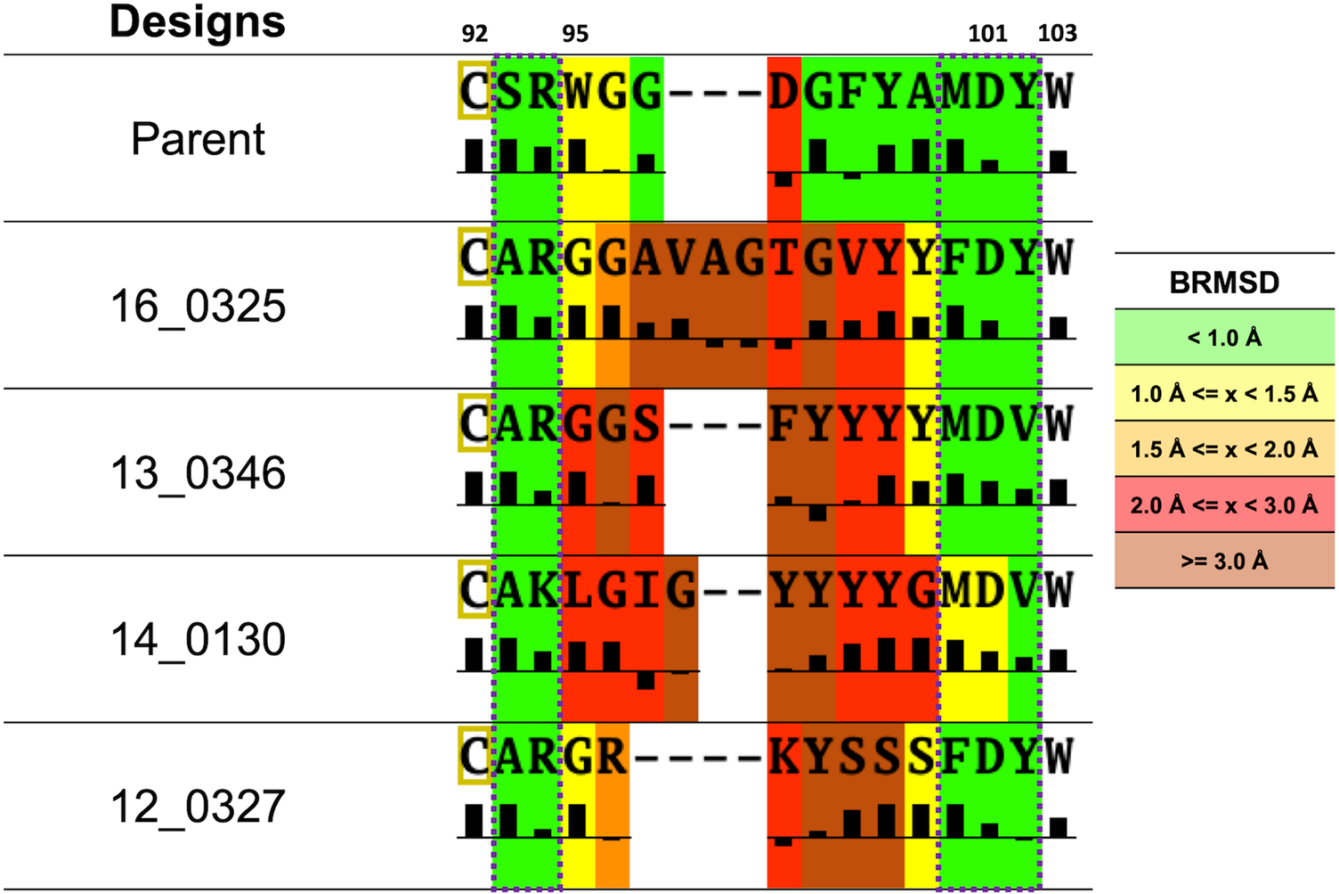
Structure prediction accuracy for the H3 designs versus surface accessibility. Backbone RMSD of the H3 loop between predicted designs and their crystal structures is highlighted by color ranges. Surface Accessibility as defined in MOE (Chemical Computing Group) is calculated using the crystal structure and is shown under each corresponding H3 loop sequence as histograms indicating the degree of burial. Positive bars indicate that a residue is buried. Dashed purple rectangles highlight H3 stem regions.

Overall, the H3 germline designs bind the same epitope as the parental bH1 Fab, with very little structural changes other than in the region directly around the H3. When comparing these designs to the parental antibody, there is 0.5–1.2 Å BRMSD movement in the backbone of the canonical loops (L1, L2, L3, H1, H2) and the Fv framework (FR), well within the resolution of the crystal structure. On the antigen side, similar to the antibody, when comparing the crystal structures of the 4 designed variants to the parental structure, little movement is seen other than in the region close to the H3. In particular, antigen residues 83–90, 83′–90′ (IKPHQGQH), which do not change conformation, are bent slightly to compensate for changes in H3 length, with the extreme case being design 13_0346 which has a BRMSD for the antigen of 2.62 Å (Table 3). The H3 loop was predicted with varying accuracy, with the best being the parental sequence with the BRMSD of 0.83 Å which benefitted from the cognate coordinates for the antigen and the rest of the Fv being used, while the prospective designs ranged from 2.13 to 3.43 Å (Table 3; Fig. 6). While BRMSD gives an overall picture of how well the predicted loop conformation compares to the crystal structure, much of the deviation occurs in the solvent exposed region/tip of the loop, which does not interact with either the antigen or antibody (Fig. 7).

The RMSDs for the modelled conformations of the four designed H3 sequences are larger than that obtained for the modelled parental sequence (Table 3). This is somewhat expected because one approximation made for this study was that the conformation of the environment will not change significantly across the different H3 sequences. In modelling the parental sequence using our loop prediction protocol, the coordinates of the surrounding residues were taken from the 3BDY crystal structure, the cognate environment of the parental H3 sequence. This likely improved the accuracy of the modelled structure of the parental sequence. To assess the effect of conformational changes in the surrounding residues, the loop ensemble was regenerated using the respective crystal structure environment instead of using the crystal structure of the parental antibody as the template for each of the designs. When this is done, the results do improve in some cases and approach the RMSD values seen for parental sequence (see Table S3).

## Discussion

**Figure 8:**
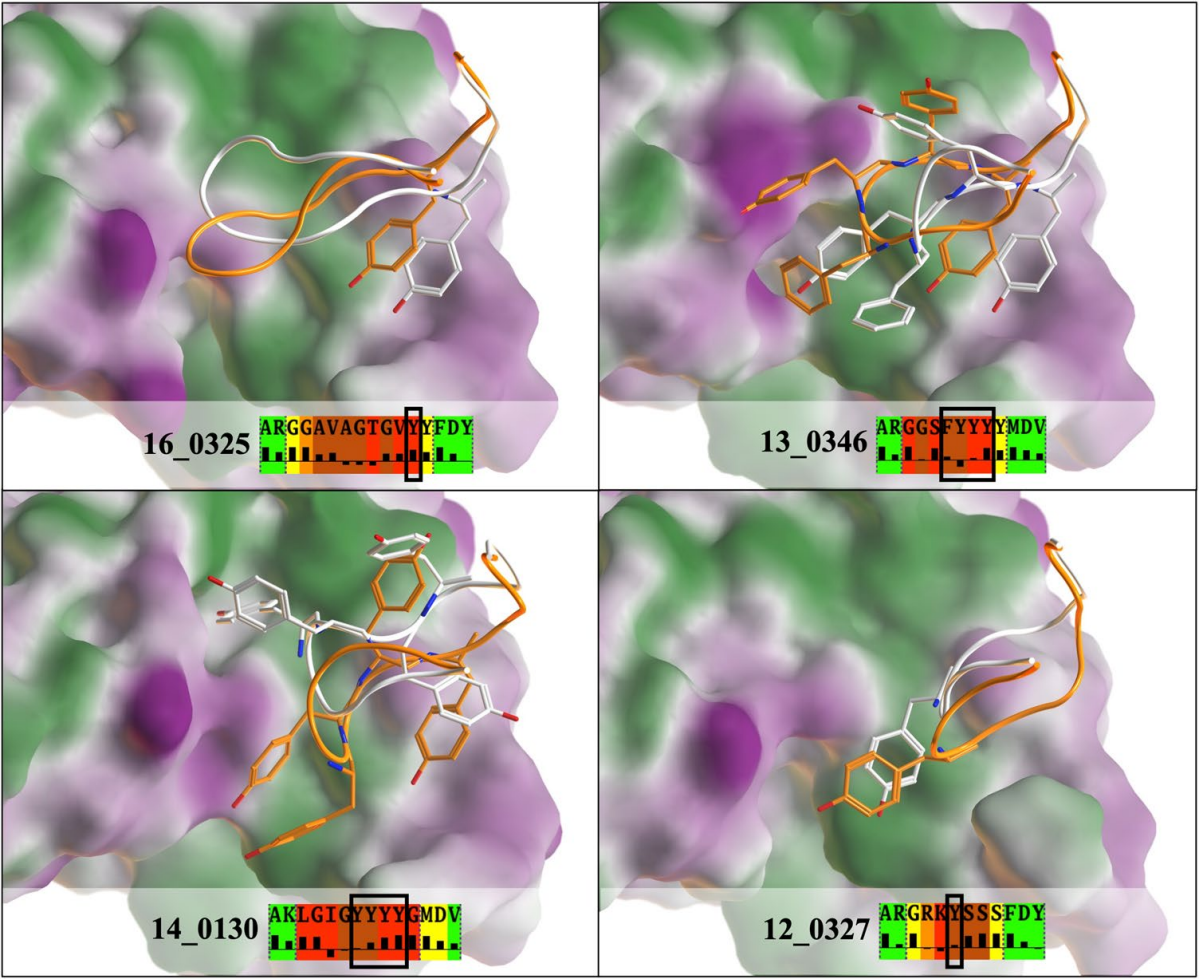
Non-polar interactions made by aromatic residues compensate for structural inaccuracies in H3 redesign. The H3 loop conformations are rendered as tubes colored white for the crystal structure and orange for the modeled structure corresponding to the lowest Talaris energy. Aromatic residues from each H3 loop boxed in the BRMSD/SASA diagrams (as per Fig. 6) are displayed with capped sticks. A molecular lipophilicity surface of the VEGF antigen is rendered for the crystal structure determined for each design (Green = Lipophilic, Purple = Hydrophilic, White = Neutral, Created in MOE).

Our antibody H3 loop screening protocol was designed to address the challenge of going beyond point mutations in redesigning H3 loops. The use of a natural repertoire of human sequences offers new opportunities for exploration of a sequence space with varying H3 loop lengths. However, this necessitated the prediction of the H3 loop conformation, which still remains a challenge with longer loops. Our modelled H3 loop conformations were not perfectly accurate, yet nanomolar affinity binders were still discovered even when the predicted H3 BRMSD values were greater than 3.0 Å relative to their crystal structures, as in the case of design 16_0325. One may ask how it was possible to discover good binders despite inaccurate prediction of the underlying structures. Upon closer comparison of the predicted and crystal loop conformations, it was found that most of the deviations occurred in the loop tips, mostly solvent exposed in the complex (Fig. 7) while the rest of the loop structures were correctly predicted. This supports the effectiveness of the H3 loop stem grafting strategy as a first step when generating an H3 loop conformational ensemble. When the H3 loop tip does interact with the antigen in the crystal structure, errors in the predicted structure are mitigated by the hydrophobic nature of many of these interactions in the H3 re-designs. Hydrophobic interactions are generally non-directional and are more tolerant of variations in the detailed positioning of the hydrophobic groups. Examples can be found in all four H3 designs that were crystallized and are illustrated in Fig. 8. In short, the imperfectly modelled structures still capture, or mimic key antibody–antigen interactions present in the true structures, which causes them to score well despite errors in the structural details.

Implied in the previous sentence is that the true conformations should score well in our protocol. To test this hypothesis, the crystal-structure conformations of the H3 loop of the 4 designs were grafted into the original parental structure (PDB ID: 3BDY) used for the initial screen. In cases where a clash with a side chain in the environment occurred with the H3, the side chain in question was mutated to the rotamer present in the respective design’s crystal structure. The H3 loop was then energy-minimized in a fixed environment and scored (“Grafted” column in Table 4). The Talaris score for the grafted crystal structure conformations are better than the predicted loop Talaris scores for all designs. The grafted crystal structure loop SIE scores are within 1.3 kcal/mol of the prediction loop SIE score, which is within the error bars of this scoring function. Our selection protocol for H3 sequences depends on the *Z*-scores. For 3 out of 4 designs, the *Z*-scores for the grafted loops are better than those of the incorrectly predicted conformations and pass the − 1.5 selection cutoff. This suggests that the *Z*-scores of our imperfectly modeled H3 loops can be viewed as upper bound scores, with the true conformations being possibly even more negative in value. It also suggests that − 1.5 is a reasonable cutoff that allows for the selection of H3 loop candidates that takes into account the less-than-ideal interactions due to errors in the structure prediction.

**Table 4:** Changes in score with variation in loop conformation and environment for the conformation with the best Talaris score.

Design ID	Talaris energy		SIE		*Z*-score	
	Predicted	Grafted	Predicted	Grafted	Predicted	Grafted
16_0325	− 278.19	− 307.63	− 14.21	− 13.66	− 1.66	− 4.58
13_0346	− 274.07	− 300.79	− 13.92	− 14.14	− 1.58	− 5.15
14_0130	− 277.26	− 308.25	− 14.96	− 13.62	− 2.21	− 4.81
12_0327	− 275.84	− 276.51	− 13.55	− 12.52	− 1.60	− 0.99

The size bias of binding affinity scoring functions is well-known in the field of small-molecule protein interactions^47–49^ and is also observed in protein–protein scoring functions. This manifests in the tendency of scoring functions to score the contributions of large amino acids such as tryptophan and phenylalanine to binding too favorably similar to the addition of large hydrophobic groups in small molecules. This scoring bias is magnified when mutations involve changes in loop length. Hence, in this study it was necessary to segregate loops according to length prior to calculation of their *Z*-scores. Otherwise, longer loops would generally outscore shorter ones and eliminate them from selection (Table S2). For example, design 12_0327 with an H3 sequence of 12 residues would not have been selected had the *Z*-scores been calculated for the entire library regardless of loop length.

Our selection of H3 loops used a consensus of *Z*-scores using the Talaris and SIE scoring functions. 11 of our 16 consensus-based designs (Table 1) fall within the top 100 of Talaris or SIE alone, indicating some overlap in the preferred sequences of the two scoring functions. However, the detailed composition and ranking of sequences in their top 100 are quite different. Moreover, the two highest-affinity designs were not in the top 100 of Talaris (16_0325: rank 153; 14_0112: rank 296), while SIE identified these two sequences in its top 15 (16_0325 : rank 13; 14_0112: rank 5). Conversely, design 16_0460 was ranked 533 by SIE but ranked 5th by Talaris. This suggests that neither scoring function can consistently rank binders well and that a consensus approach is a practical way to hedge our bets.

One concern in this study was that the remaining non-H3 CDRs would retain enough affinity such that any non-clashing H3 would exhibit binding and appear to be a successful H3 loop design. This was addressed by using the minH3 construct which uses a truncated H3 sequence, retaining only the stem residues from the parental H3 sequence bridged by a glycine, which should not directly interact with VEGF. The minH3 construct was stable and weakly binding. This provides a baseline by which to judge whether a given H3 loop sequence is actually contributing to binding. 13 of the 16 designs showed favourable contributions to binding surpassing the baseline (Table 2). Even in cases where the improvement over baseline is modest, further improvement is in principle possible using a point mutation approach such as ADAPT^18^. The combination of H3 loop replacement and subsequent point mutations with ADAPT is a potentially powerful approach for antibody redesign and discovery.

RosettaAntibodyDesign^33^ (RAbD) is similar in spirit but also orthogonal to our work. Just as in our case, RAbD draws from a database of loop sequences to use as candidates for replacing an existing loop. However, RAbD’s focus is re-designing non-H3 loops by taking advantage of their canonical conformations, while this work focused on the hypervariable H3 loop using a novel loop grafting-sampling-scoring protocol. Also, the RAbD sequence repository is drawn from known crystalized CDR loops while this work is based on a larger set of human H3 sequences not limited to crystal structure availability. Both approaches successfully designed true binders, with the best designs achieving a greater than tenfold increase in binding affinity compared to their parental antibody used as template. The two approaches are complementary and if used together may provide a comprehensive approach to CDR redesign.

In summary, this work has shown the successful re-design of the H3 loop for the bH1 antibody which targets VEGF. This was accomplished by virtually screening a small library of H3 loop sequences. It expands the sequence space search beyond that explored by point mutations of a parental structure.

## Materials and methods

### Initial structure preparation for target antibody–antigen complexes

Each antibody–antigen complex was prepared using the previously published protocol^51^. Each structure was downloaded from the PDB and the biomolecular transformation applied when present. Each antibody–antigen complex was processed with an automated preparation script to generate the initial structures. The main steps in the preparation are listed below:

1. Retain only first refined copy,
2. Delete water, metals and halogen ions,
3. Assign Kabat numbering using AbNum^52^,
4. Addition of disulfide bonds,
5. Addition of missing side chain atoms (no repacking),
6. Capping of chain breaks,
7. Capping/charging chain termini (capping was done when there was a difference in sequence between the atom and sequence recorded of the PDB file),
8. Addition of missing hydrogens and assignment of protonation states (pH 7) and optimization of hydrogen bond network using an in-house program,
9. AMBER energy minimization of added hydrogen atoms and any newly added side chain atoms and capping groups with harmonic restraints on all the other heavy atoms of 1000 kcal/mol/Å^2^,
10. AMBER energy minimization of the entire complex, with a 10 kcal/mol/Å^2^ harmonic restraint applied to backbone heavy atoms, a 1 kcal/mol/Å^2^ harmonic restraint applied to side chain heavy atoms while all hydrogens are free to move.

### Grafting stems into antibody–antigen complex

A library of stem templates was created to simplify the grafting of the stems into the target structure by creating all loop lengths for a given stem cluster. Stem templates were created by starting from the representative PDB structure identified by North et al.^45^ and using a poly-glycine linker to complete the loop. This was done for all stem clusters for H3 loop lengths of 7–30 residues according to IGMT definition^46^ to create a library of stem clusters for a series of H3 lengths. These new H3 loop templates were all placed into the 3BDY framework.

For each new H3 sequence, matching stems templates were found by aligning to stem sequences using a hidden Markov model and selecting the template with the appropriate loop length. When the alignment score of multiple stems was within 1.7 units of the best match, all stem templates were retained and grafted into the target, referred to as the stem target.

To graft the stems template into the appropriate target framework CA atoms of residues H90-92 and H103-105 between the template and the target antibody–antigen complex were superposed using the McLachlan algorithm^53^ as implemented in the program ProFitv3.1 (Martin, A.C.R., http://www.bioinf.org.uk/software/profit/). Residues H90 to H105 were copied from the stem templates into the target complex. Once inserted, the antigen was ignored to ensure that H3 loop maintained reasonable bond lengths, angles and overall conformation during the following steps. SCWRL4^54^ was used to mutate the copied residues to their correct sequence. Residues H90-H105 underwent a restrained energy minimization using AMBER FF99SB^55^ using a distance dependent dielectric of 4, a 5 kcal/mol/Å^2^ harmonic restraint on the CA, N, C, O backbone atoms and allowing Hs and side chain atoms to be free with all other atoms being fixed.

### Generating H3 loop ensembles

A major challenge in this study was the number of H3 loop sequences to be modelled. To achieve this goal a hierarchal rigid approach was developed to generate a conformational ensemble. Using the default and published Rosetta approach would have required a significant amount of computational time for modelling 5000 loops. To decrease the time, a fast conformational search was done outside Rosetta to generate an initial set of loop conformations. This ensemble was then sent to Rosetta for refinement and enhanced scoring. The details of the method are described below.

An initial fast conformational search is done using Loopy^56,57^, which is used to create a large ensemble of diverse conformations. If multiple grafted stems targets were created in the previous step, they were treated as distinct runs and merged prior to the refinement stage. The first step of the initial ensemble generation was creating 5000 H3 loop inner-tip conformations using Loopy^56,57^ with default parameters and the loop tip (the part being searched) defined based on loop length (Table 5). The loop tip definition varied to ensure that a minimum of 5-residue loop was searched when the H3 was 8 residues or longer. For loops of length 7, the work of North et al*.*^45^ identified two conformations for 7 residues and therefore the tip did not need to be searched. For loops shorter than 7 residues, stem clusters were not identified necessitating the entire loop to be searched. Once an ensemble has been generated by Loopy, the first 1000 conformations were scored and re-ranked using dDFIREv1.1^58,59^ with default settings, similar in spirit to LoopBuilder^56^. The top 250 conformations according to dDFIRE v1.1 were then filtered to ensure they have a minimum number of contacts with the antigen, thus ensuring that the H3 contributes to binding. The minimum number of contacts was defined as the median number of contacts of the top-1000 Loopy conformations. A contact was defined as any heavy atom between the antibody and antigen within 4.5 Å. On average, 50% of the loop conformations were removed. These loop conformations then had their hydrogen-bond network optimized using an in-house tool^51^ followed by an energy minimization with AMBER FF99SB^55^ using a distance-dependent dielectric of 4, with the H3 loop atoms free to move and all other atoms fixed. The top-100 conformations per H3 stem according to their AMBER energy were retained. For example, if an H3 sequence had 2 stem templates, 200 loop conformations were retrained; and for 4 stem templates, 400 loop conformations were retained. Absolute scores between the two ensembles for the same sequence are not directly comparable due to the different stem conformations (which were not included in the search) putting them in different energetic reference frames.

**Table 5:** Loop tip definition used for conformational ensemble generation with Loopy.

H3 length (aa)	Loop tip definition
6	Entire loop
7	No loop tip
8	H93 + 1 to H102 − 2
9	H93 + 2 to H102 − 2
≥ 10	H93 + 2 to H102 − 3

To put all ensembles into the same energetic frame of reference, the entire H3 loop, not just the loop tip, was refined using 5 steps of Rosetta KIC refinement^60^ on the best 100 conformations per ensemble according to AMBER energy. Only the H3 loop, as defined at H93 to H102 is refined using the following command:

- $ROSBIN/loopmodel.linuxgccrelease -database path_to_rosetta_database \
  -in::file:fullatom -loops::loop_file loop_defn.txt \
  -in:file:s input_ros.pdb -loops::remodel no -loops::refine refine_kic -kic_rama2b \
  -loops:ramp_fa_rep -loops:ramp_rama -kic_omega_sampling \
  -allow_omega_move true
  -loops:refine_outer_cycles 1 -loops:max_inner_cycles 10 \
  -loops:neighbor_dist 0.0 -ex1 -ex2 -talaris2014 true \
  -nstruct 5

Following initial refinement, if multiple stem targets where used, all results were merged and only the loop conformations for a given sequence within 5 kcal/mol of the best-scoring one according to Talaris 2014 energy were retained. The top-scoring loops then underwent a secondary 25-step Rosetta KIC refinement using the same commands shown above except for nstruct = 25 for expanding the ensemble around these low-energy conformations. As before, only the loop conformations for a given sequence within 5 kcal/mol of the best-scoring conformation according to Talaris 2014 energy were retained. This ensures a dense ensemble around the best-scoring conformation according to Rosetta’s Talaris scoring function.

We note that the entire protocol is based on a rigid structural context surrounding the H3 loop. This is required for speed and accuracy in affinity scoring, even if the underlying structure may not reflect reality and may have been compromised locally. The errors resulting from the structural inaccuracies due to rigid context are thought to be smaller than those due to the noise introduced when treating flexibility explicitly^61^.

The above steps for conformational sampling of H3 loop sequences is summarized in Figure S3.

### Scoring and selecting candidate sequences

Once the ensemble is generated, a median-based composite *Z*-score is calculated by using Boltzmann averaging of the Talaris 2014 energy and SIE affinity scores. The median value used to calculate the *Z*-score is evaluated for each H3 loop length independently unless otherwise noted. The Talaris 2014 energy score was taken directly from the output of the loop ensemble generated previously. SIE was calculated after the hydrogen-bond network was optimized using an internal tool^51^ and the H3 loop was energy-minimized with AMBER FF99SB^55^ using a distance-dependent dielectric of 4, while its environment was fixed, and with the H3 loop heavy atoms restrained with a harmonic force constant of 1 kcal/mol/Å^2^.

Following the calculation of the *Z*-score, H3 sequences were selected if they met the following criteria. First, an accepted sequence must have a *Z*-score of − 1.5 or less. Secondly, H3 loop sequences having best-scoring conformation according to Talaris energy that place any residues in the disallowed region of the Ramachandran phi-psi backbone dihedral angles were discarded. This was done using ProCheck^50^ on an extracted loop segment comprised of the residues H92 to H103. Third, accepted H3 sequences must have a similar or better hydrogen-bond score than the parental H3 sequence. This was calculated as the sum of intermolecular HB-energy and HB-flaw terms from ProPOSE^51^ using the best-scoring loop conformation according to Talaris energy. Any sequence with an HB-energy or HB-flaw term greater than + 1 unit than the parental bH1 antibody sequences was eliminated. The remaining loops then underwent a visual inspection to select the final candidates for experimental validation. We focused on three issues during this final processing. First, we ensured selected sequences retain at least some contacts seen in the parental H3 sequence. Secondly, we ensured that, if present, the H94 Arg side chain maintains an electrostatic interaction with H101 Asp side-chain in the stem regions. Lastly, we ensured that no charges were buried by the grafted H3 loop without establishing favorable electrostatic contacts. The H3 sequences which did not follow these three criteria were eliminated. Typically, conformations which preserve similar interactions as the parental structure were retained. While visual inspection is admittedly subjective, it is still a key step in any computational screen^18^. To partially demystify these subjective decisions, it is important to document the rationale behind them, as done above.

### Grafting crystal loop conformations for retrospective analysis

The grafting of the crystallographic H3 loop conformations for the 4 crystalized designs onto the parental structure was completed by first superposing the crystal structures of the 4 design onto the parental bH1-VEGF co-crystal structure (PDB ID: 3BDY) which was used as a template for H3 loop screening. Superposition was done in MOE2019.01 (Chemical Computing Group) using the backbone atoms of the antibody framework and default options. The H3 loops (residues H93 to H102) of the 4 designs were then grafted into 3BDY using MOE 2019.01 Loop Graft feature. The following rotamers were copied from the designed crystal structure to ensure the grafted loop does not clash with the environment:

- 16_0325, Antigen residue 48 rotamer was copied from crystal into parent
- 13_0346, H52 rotamer was copied from crystal into parent
- 14_0130, Antigen residue 48 rotamer was copied from crystal into parent
- 12_0327, L91 rotamer was copied from crystal into parent

Single-structure SIE values were calculated in the same manner as during loop selection (see above) following the same energy minimization protocol. The Talaris energy was calculated following energy-minimization with Rosetta using the following command:

- $ROSBIN/minimize.default.linuxgccrelease \
  -in:file:s ${oroot}_inp.pdb \
  -run:min_type lbfgs_armijo_nonmonotone -run:min_tolerance 0.001 \
  -movemap ${oroot}.map -overwrite \
  -corrections::restore_talaris_behavior \
  -score:weights talaris2014

The Talaris score was taken from the energy minimization output. These single-structure values were then used to calculate the *Z*-scores for each of the grafted designs by employing the median values previously calculated during the H3 loop screening (see above).

### Protein production

Production of the constructs is as described previously^62^. cDNA for the heavy and light chains of Fab variants of bH1 were ordered from commercial vendors (Thermo-Fisher/Life Technologies Inc., Burlington, ON, Canada; GENEART, Regensburg, Germany). These contained signal peptide sequences, and heavy-chain C-terminal His_8_ tags. Productions were carried out by co-transfection of CHO-3E7 cells^63^ at various scales between 200 mL and 1 L. Transfections were performed at a cell density between 1.8 × 10^6^ to 2.0 × 10^6^ cells/mL with viability greater than 98%. Cells were distributed in 1.0–2.8 L-shaker flasks and transfected with 1 μg of total DNA per 1 mL of production [50% of total DNA contained heavy chain and light chain constructs at ratios of 1:1 (w/w)] using PEI MAX™ (Polysciences, Inc., Warrington, PA). The final DNA: PEI MAX™ ratio was 1:4 (w/w). Cell cultures were incubated for 24 h on an orbital shaking platform at an agitation rate of 110 rpm at 37 °C in a humidified 5% CO_2_ atmosphere. Twenty-four hours later, the cultures were fed with Tryptone N1 at 1% w/v final and Valproic acid sodium salt at 0.5 mM final concentration and transferred to 32 °C for 6 days. Cell density and cell viability were determined by direct counting of cell samples with a Vi-CELL automated cell counting system (Beckman Coulter Life Sciences, Indianapolis, IN) using the trypan blue dye exclusion method.

### Protein purification

Purifications from cell-culture supernatants were performed by immobilized metal-affinity chromatography for all Fab variants as described previously^62^. Fab samples were loaded onto a 1 mL HisTrap™ Excel column (GE Healthcare Life Sciences, Uppsala, Sweden) equilibrated in HyClone™ Dulbecco's phosphate-buffered saline (DPBS). The column was washed with DPBS and Fabs were eluted with 500 mM imidazole in DPBS. Fractions containing the Fabs were pooled and the imidazole buffer was exchanged against DPBS on PD10 columns (GE Healthcare Life Sciences). Purified Fabs were aseptically filtered through 0.2 μm filters. All affinity purified samples were further purified by preparative SEC on Superdex-200 pg columns (GE Healthcare Life Sciences). Selected peak fractions were concentrated by ultrafiltration using Vivaspin^®^ 6 centrifugal concentrators with a membrane molecular weight cut off of 10 kDa (GE Healthcare Life Sciences) at 15 °C following the manufacturer’s instructions. During the process, the protein concentration was monitored on a NanoDrop™ 2000 spectrophotometer (Thermo Fisher Scientific, Waltham, MA) using absorbance at 280 nm and the calculated specific extinction coefficient of each variant.

### Differential scanning calorimetry

DSC was used to determine the thermal transition midpoint (*T*_m_) of selected Fab variants as previously performed^62^. DSC experiments were run using a VP-Capillary DSC system (Malvern Instruments Ltd, Malvern, UK). Samples were diluted in HyClone™ Dulbecco's phosphate-buffered saline (DPBS; GE Healthcare Life Sciences) to a final concentration of 0.4 mg/mL. Thermal denaturation was carried out under 70 psi of nitrogen pressure by increasing the temperature from 20 to 100 °C at a rate of 60 °C/h, with feedback mode/gain set at “low”, filtering period of 8 s, prescan time of 3 min. The experiment was run three times with the parental Fab as reference in order to report the precision of the method, but only one time for each other sample. All data were analyzed with Origin 7.0 software (OriginLab Corporation, Northampton, MA). Thermograms were corrected by subtraction of corresponding buffer blank scans and normalized to the protein molar concentration. The *T*_m_ were determined using the automated data processing with the rectangular peak finder algorithm for *T*_m_.

### Surface plasmon resonance

Full-length isoform 165 of human VEGF-A (termed VEGF here) was produced recombinantly in HEK and purified. SPR binding assays were carried out on a Biacore T200 instrument (Cytiva Inc., Vancouver, BC) at 25 °C using PBS running buffer containing 0.05% Tween 20 (Teknova, Hollister, CA) with the addition of 3.4 mM EDTA. VEGF-A or TGFb-2 surfaces and a paired matching mock-activated blank surfaces were prepared on a CM-5 sensorchip using standard amine coupling with 10 mM Na acetate buffer pH4.5 for immobilization. To analyze binding, each Fab variant was injected using single cycle kinetics using five increasing Fab concentrations optimized for the variant’s affinity, with an association of 150–180 s, and dissociation phase of 150–1200 s at a flow rate of 50 μL/min. For truncated Fab variants, the association phase was reduced to 30 s at a flow rate of 20 μL/min to minimize sample consumption. Each Fab variant was analyzed with three independent injections except for H3-less Fabs with no binding activity. The resulting sensorgrams were double referenced and analyzed for binding kinetics by fitting to a 1:1 Langmuir binding model in Biacore T200 Evaluation Software v3.0 (Cytiva Inc., Vancouver, BC).

## Supplementary Information

Supplementary Information.
