# Supplementary material for: Redesigning an antibody H3 loop by virtual screening of a small library of human germline-derived sequences
Source: Sci Rep. 2021 Nov 1;11:21362. doi: 10.1038/s41598-021-00669-w (PMC8560851; doi:10.1038/s41598-021-00669-w)
Supplement: Supplementary file 1 — Supplementary Information. [file 41598_2021_669_MOESM1_ESM.pdf]

Supplementary Information for:

Redesigning an antibody H3 loop by virtual screening of a small library of human germline-derived sequences

Christopher R. Corbeil[1], Mahder Seifu Manenda[2], Traian Sulea[1], Jason Baardsnes[1], Marie-Ève Picard[2], Hervé Hogues[1], Francis Gaudreault[1], Christophe Deprez[1], Rong Shi[2], and Enrico O. Purisima[1,3,*]

[1] *Human Health Therapeutics Research Centre, National Research Council Canada, 6100 Royalmount Avenue, Montreal, QC, Canada H4P 2R2*

[2] *Département de Biochimie, de Microbiologie et de Bio-informatique, PROTEO, and Institut de Biologie Intégrative et des Systèmes (IBIS), Université Laval, Pavillon Charles-Eugène-Marchand, Québec City, QC, Canada G1V 0A6*

[3] *Biochemistry Department, McGill University, 3655 Promenade Sir William Osler, Montréal, QC, Canada H3G 1Y6*

* Enrico.Purisima@nrc-cnrc.gc.ca

Supplementary Tables and Figures

Table S1. Backbone RMSD vs Stem Residue Position for 248 Antibody:Antigen complexes. The results have been broken down by ranges of loop length. 7 to 24 corresponds to all loops in the dataset. Loop length 7 is separated out as there are only two distinct loop conformations according to North *et al.*[1] and therefore no loop search is needed. 8 to 11 corresponds to loops on the shorter side of the germline library (25%). 12 to 13 correspond to medium size loops in the germline library (27%) where the loop tip to be searched is 7 to 8 residues long and overall good prediction would be expected with published loop methods. 14 to 16 corresponds to the longest loop in the germline library (46%) and corresponds to loop tips of 9-11 residues which may pose some difficulty for most loop prediction methods. Cells in orange correspond to RMSD values greater than 0.75Å.

Loop Length		BMRSD Å (N, CA, C, O)						
min	max	H93	H93 + 1	H93 + 2	H102 - 3	H102 -2	H102 - 1	H102
7	24	0.30	0.48	1.40	1.06	0.63	0.56	0.41
7	7	0.28	0.42	0.40	0.48	0.37	0.44	0.40
8	11	0.33	0.49	1.45	1.25	0.73	0.58	0.43
12	13	0.29	0.51	1.45	0.91	0.56	0.52	0.40
14	16	0.29	0.38	1.37	0.82	0.56	0.62	0.41

Table S2. Number of CDR-H3 loop sequence with Z-Scores lower than –1.5.

	N (Z-Score <= –1.5)	
H3 Length	By H3 Length[a]	Complete[b]
7	2	0
8	3	0
9	5	0
10	6	0
11	3	0
12	3	0
13	13	7
14	22	33
15	20	36
16	11	37

[a] The Z-Score is calculated using only sequences of a given H3 loop length.
[b] The Z-Score is calculated using sequences of all H3 loop lengths.

Table S3. Comparison of CDR loop prediction accuracy between using the parent 3BDY crystal structure versus using the cognate crystal structure for the surrounding residues.

Design	3BDY as Starting Template			Crystal Structure as Template		
	**Top 1**	**5 kcal/mol**	**Closest**	**Top 1**	**5 kcal/mol**	**Closest**
Parent	1.47	1.22	1.22	1.47	1.22	1.22
16_0325	3.21	2.74	2.55	3.69	2.80	2.12
14_0112	3.12	2.76	1.44	0.92	0.59	0.59
14_0472	5.95	5.93	5.47	1.43	1.35	1.35
13_0346	2.58	2.25	2.24	2.65	1.97	1.97
Avg of Designs	*3.71*	*3.42*	*2.93*	*2.17*	*1.68*	*1.51*
Avg for 14-16 CDR-H3 Length				2.26	1.66	1.56
**BRMSD**						
**Design**	**3BDY as Starting Template**			**Crystal Structure as Template**		
	**Top 1**	**5 kcal/mol**	**Closest**	**Top 1**	**5 kcal/mol**	**Closest**
Parent	0.83	0.61	0.61	0.83	0.61	0.61
16_0325	3.04	2.32	2.12	3.36	2.67	2.03
14_0112	2.61	2.49	1.01	0.70	0.46	0.46
14_0472	3.43	3.40	3.34	0.59	0.48	0.48
13_0346	*2.13*	1.80	1.78	2.24	1.52	1.52
Avg of Designs	*2.80*	*2.50*	*2.06*	*1.72*	*1.28*	*1.12*
Avg for 14-16 CDR-H3 Length				1.48	1.02	0.92

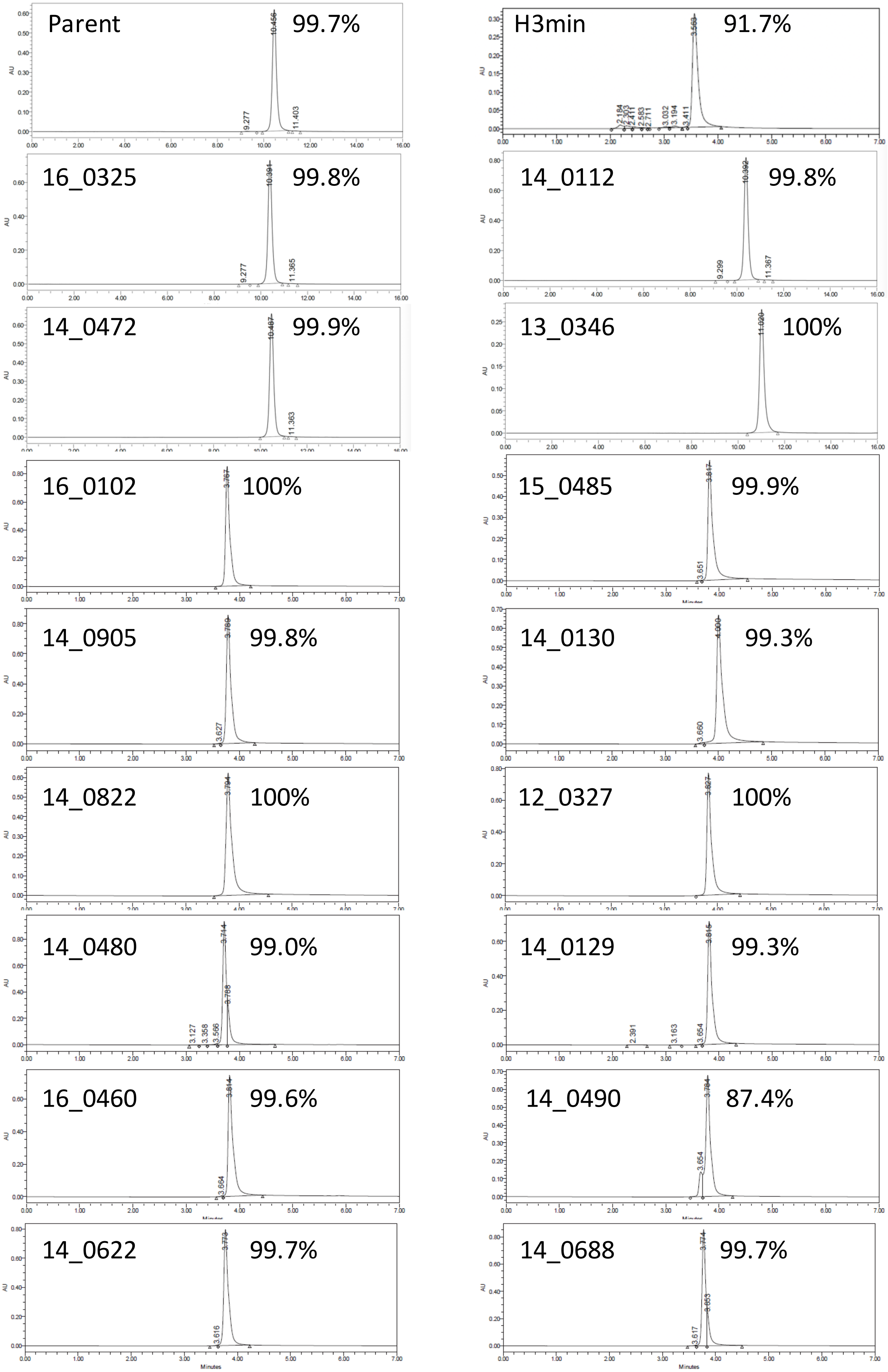
Parent 99.7%
H3min 91.7%
16_0325 99.8%
14_0112 99.8%
14_0472 99.9%
13_0346 100%
16_0102 100%
15_0485 99.9%
14_0905 99.8%
14_0130 99.3%
14_0822 100%
12_0327 100%
14_0480 99.0%
14_0129 99.3%
16_0460 99.6%
14_0490 87.4%
14_0622 99.7%
14_0688 99.7%
AU
Minutes

Figure S1. UPLC-SEC chromatograms of the parental and H3-redesigned Fab variants after IMAC purification (see Materials and Methods section). Main peak contribution is indicated.

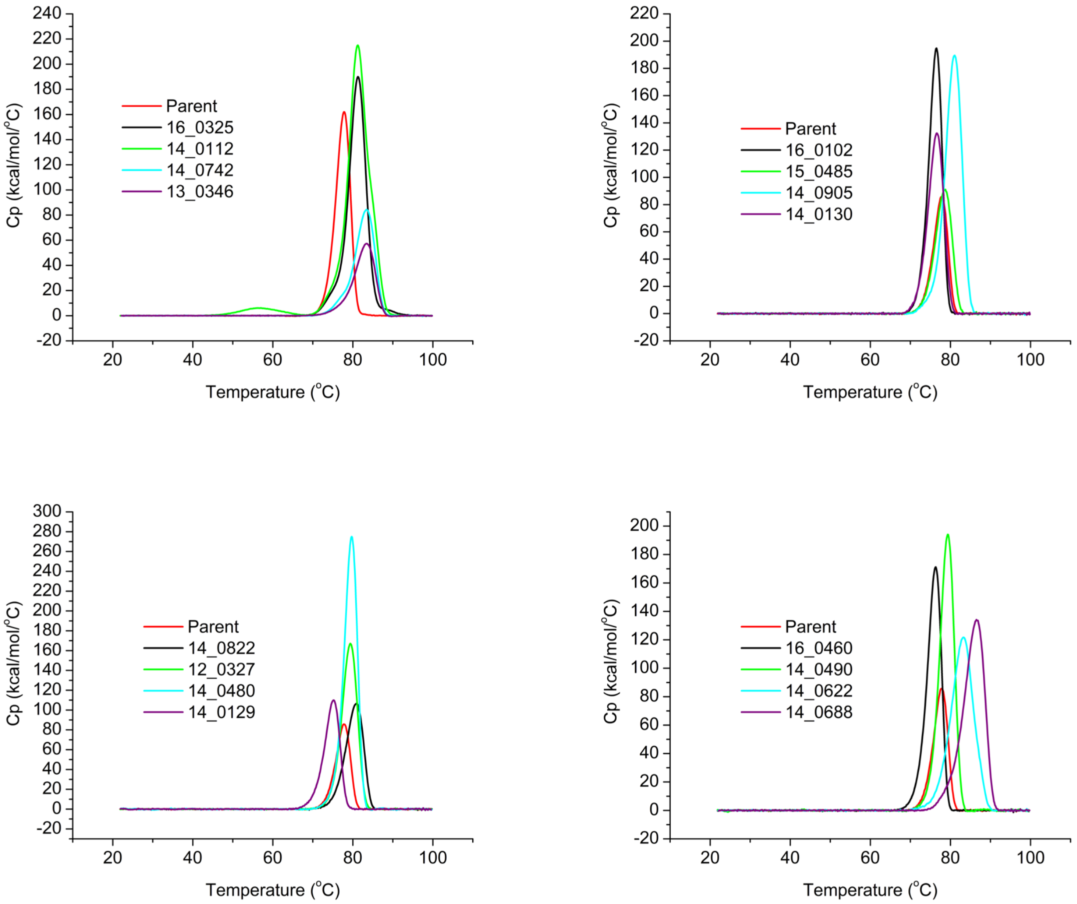

Figure S2. DSC thermograms of the variants relative to the parental Fab. See the methods for experimental details.

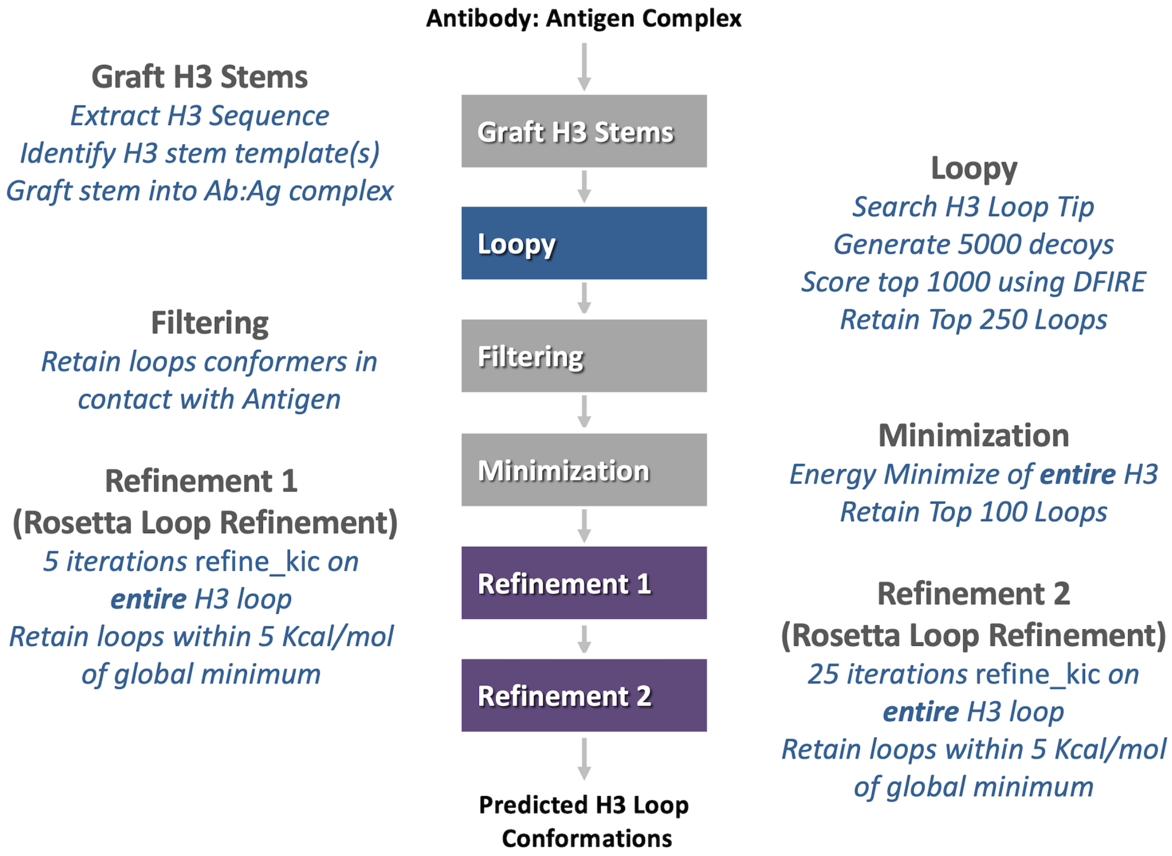

Figure S3. Conformational sampling of grafted H3 loops. With the stem regions grafted into the parent antibody-antigen structure, a hierarchal approach to conformational sampling of the inner tip of the loop was created. If the H3 sequence fits into multiple stem templates, both stems are included in the search protocol. Initially, Loopy is used to quickly generate inner-tip conformations, which are then rescored using DFIRE and filtered to identify loop conformation which are in contact with the antigen. These loops are then energy-minimized with AMBER and go through a two-stage refinement using Rosetta KIC method to generate a dense ensemble around the best-scoring conformation for further affinity scoring.

References

1. North, B.; Lehmann, A.; Dunbrack, R. L., A New Clustering of Antibody CDR Loop Conformations. *J. Mol. Biol.* **2011,** *406* (2), 228-256.
